# The tumor microenvironment across four dimensions: assessing space and time in cancer biology

**DOI:** 10.3389/fimmu.2025.1554114

**Published:** 2025-06-23

**Authors:** Christina R. Larson, Ayushi Mandloi, Satwik Acharyya, Julienne L. Carstens

**Affiliations:** ^1^ Department of Medicine, Division of Hematology and Oncology, O’Neal Comprehensive Cancer Center, Immunology Institute, University of Alabama at Birmingham, Birmingham, AL, United States; ^2^ Department of Biostatistics, University of Alabama at Birmingham, Birmingham, AL, United States; ^3^ Department of Biomedical Informatics and Data Science, University of Alabama at Birmingham, Birmingham, AL, United States

**Keywords:** spatial omics, temporal analysis, tumor microenvironment, tumor immune microenvironment, tumor evolution, spatiotemporal, intravital imaging

## Abstract

The tumor microenvironment is heterogeneous, structurally complex, and continually evolving, making it difficult to fully capture. Common dissociative techniques thoroughly characterize the heterogeneity of cellular populations but lack structural context. The recent boom in spatial analyses has exponentially accelerated our understanding of the structural complexity of these cellular populations. However, to understand the dynamics of cancer pathogenesis, we must assess this heterogeneity across space and time. In this review, we provide an overview of current dissociative, spatial, and temporal analysis strategies in addition to existing and prospective spatiotemporal techniques to illustrate how understanding the tumor microenvironment, focusing on dynamic immune-cancer cell interactions, across four dimensions will advance cancer research and its diagnostic and therapeutic applications.

## Introduction

A common storytelling technique comes from the Latin phrase *in medias res*, meaning “in the middle of things.” This tool captures the audience’s attention by starting in the middle or climax of the narrative then backing up to explain how the story reached that point. Similarly, a scientist’s attention is often captured by an interesting phenotype, causing them to wonder, “how did that end up there.” Just as flashbacks reveal plot while describing characters, setting, and conflict to catch the audience up with the story, experiments reveal important cell types, cell states, and signaling pathways to help scientists piece together their own narrative. Dissociative techniques, such as flow cytometry and single cell transcriptomics, have enabled the identification of the array of characters in these stories and sometimes their function at the point of analysis. The rapid expansion of spatial biology and spatial multi-omics has helped describe setting and neighborhoods, which could influence the character’s function. However, to understand the full plot of a research narrative, we need to study the changes and effects of those conditions over time, not only in the middle of things, in the moment of the assay. This is especially important in complex, heterogeneous systems like tumor microenvironments (TME).

TME have been profiled across cancer types and are each uniquely challenging to treat. Some prove more challenging than others due to heterogeneity in TME composition, stromal barriers, and enhanced immunosuppression. TME have been extensively studied across the cancer biology field, but these studies largely assay TME components in isolation. By studying TME in the context of space and time (four dimensions), a more complete narrative of each cancer can be written. Patients could be better classified into responders and non-responders for specific therapies. Scientists could identify when and where a target is most vulnerable to manipulation and which cells are nearby to assist in the targeting. Understanding the spatiotemporal changes of a TME would improve biopsy analysis to advance patient therapy and outcome.

**Figure 1:**
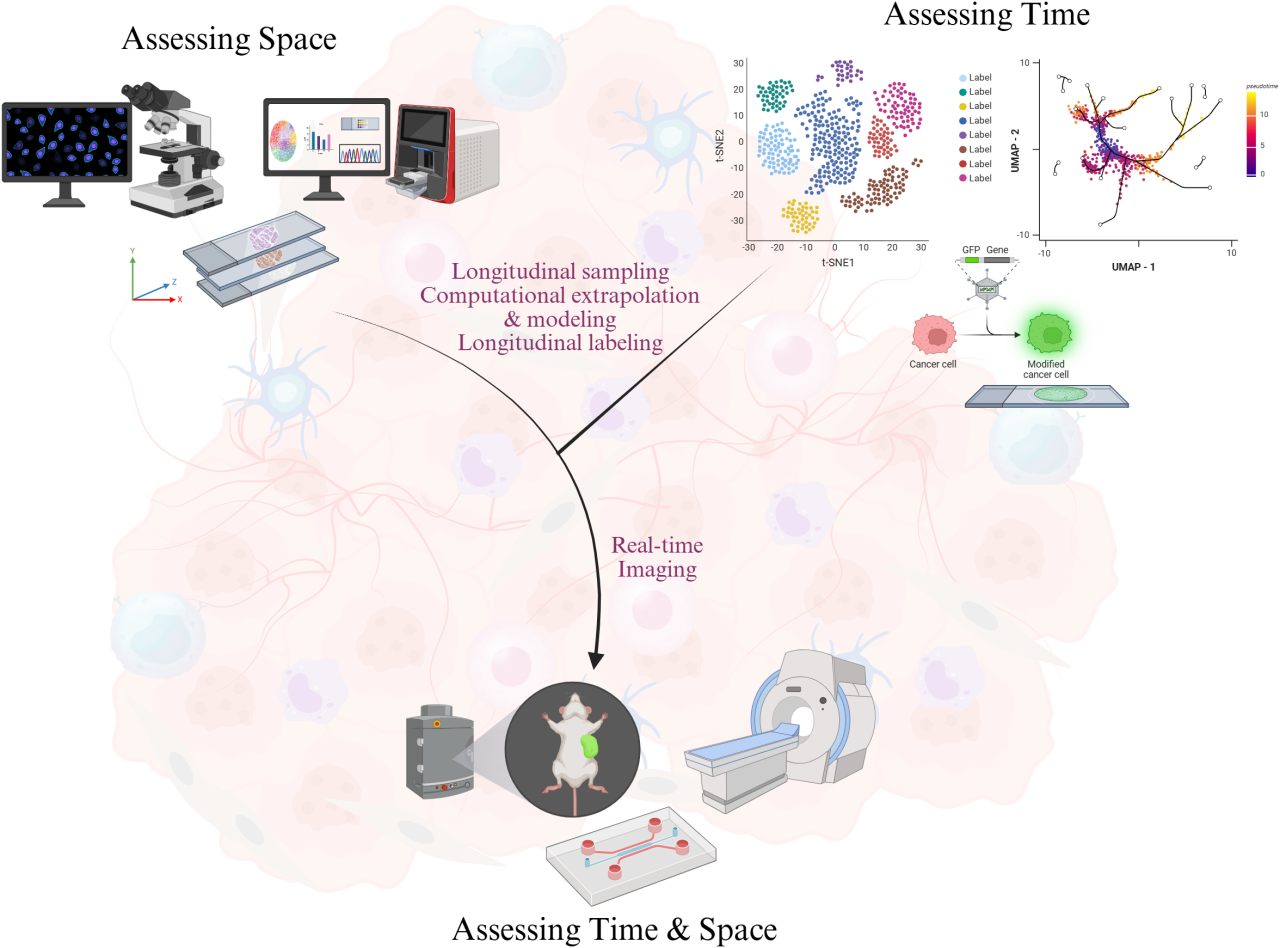
Integrating space and time in the study of the tumor microenvironment. Assessment of time is performed using four overlapping techniques: (1) longitudinal sampling, (2) longitudinal labeling, (3) computational extrapolation, and (4) live imaging. Incorporating spatial quantifications with the temporal assessments can inform tumor evolution and dynamics. Portions of this figure were created in BioRender.com. Mandloi, A (2025). https://BioRender.com/f77v802.

Accordingly, recent advances in bench methodology have expanded TME analysis to include spatial and temporal methods. The spatial platforms we will discuss have helped characterize cell localization within the TME, but many of these modalities fail to demonstrate how these cell types, especially immune cells, and the tumor change over time. Temporal strategies like Zman-seq and pulse-chase enabled by computational approaches such as TDEseq and PseudotimeDE have shown the dynamics of molecule secretion and cellular infiltration in the TME (1, 117). Although these temporal tools were developed in isolation of space, their integration with spatial methodologies cannot be dismissed. In this review, we discuss how temporal dimensions can be incorporated into existing methods through longitudinal sampling, computational extrapolation and modeling, and longitudinal labeling. Longitudinal sampling provides insights into developmental processes and tumor progression based on the assumption that chances of particular cellular dynamics are consistent between samples while longitudinal labeling identifies specific molecules or cells at one time point and assesses the changes at later time points. Real-time imaging already combines longitudinal sampling and longitudinal tracing to record biological processes as they occur, and clinical imaging allows clinicians to monitor temporal changes within the TME (**Figure 1**). Nevertheless, spatiotemporal analysis represents the next frontier in TME research. The innovations we propose are initial steps towards transitioning from *in medias res* perspective to a more complete narrative of biological phenomena.

## Assessing identity

A cell's identity is determined by its distinct transcriptional profiles and corresponding proteomic output**s**, which collectively govern its functional characteristics and phenotypic behavior within a given tissue or organism (1). One such method that enables researchers to explore the transcriptome at the level of individual cells is single-cell RNA sequencing (scRNA-seq), providing a high-resolution view of cellular identity, serving as a valuable tool to characterize cellular heterogeneity (2, 3). It is routinely used to capture the transcriptome of a single cell; however, ongoing advances have enabled nuclear transcriptome profiling using single-nuclear RNA-seq (snRNA-seq), which profiles transcriptomes in cells that are hard to dissociate. A study by Liu et al. used snRNA-seq to reveal distinct temporal shifts in the cervical cancer TME from stage-I to stage-II, showing an immune-active environment with proinflammatory macrophages and activated CD8^+^ T cells in stage-I, versus an immunosuppressive, growth-focused TME with fewer immune cells, more collagen, and increased mitochondrial activity in stage-II. This study indicates how changes in TME fuel cancer progression and point to stage-specific treatment possibilities (195). Another multi-omics technique called single-cell Assay for Transposase-Accessible Chromatin sequencing (scATAC-seq) is employed to study chromatin accessibility and characterization of regulatory elements. It is often used in combination with scRNA-seq to explore cancer-specific traits, linking chromatin accessibility changes to gene expression (5). scATAC-seq profiling of the TME in basal cell carcinoma revealed regulatory networks in immune, stromal, and cancer cells, identifying mechanisms behind T cell exhaustion in tumor-infiltrating lymphocytes (6). Among other innovations, tools like Perturb-seq track responses to genetic changes while PROFIT-seq studies other RNA molecules, enhancing our understanding of the TME (4, 7–12). Overall, advances in distinguishing cellular identities and functions have significantly improved our understanding of the characters in the tumor progression narrative, but these methods are only single dimension assessments.

## Assessing space

Space serves as a foundational concept for understanding the structure and location of biology. At its core, spatial data preserve the x, y (2D) and, sometimes, z (3D) dimensions and provide details of cellular components and structural organization, enabling a comprehensive view into the spatial relationships within complex systems. Cellular neighborhoods and spatial organization can impact clinical outcomes and can even serve as prognostic markers (13–17). This spatial assessment ranges from simple chromogenic microscopy of digital pathology to the rapidly progressing field of spatial multiplexing and omics, which has been extensively reviewed elsewhere and only briefly discussed here (18–23).

Popular spatial proteomics techniques often involve the hyper-plexing of many antibodies to detect protein targets, but they can also use mass spectrometry imaging for many molecules, including proteins, metabolites, sugars, and metals (24–26). Various academic and commercially available platforms have been used to map the protein and cellular localizations within tissues, providing insights into the complex organization and dynamics of proteins in cancerous tissues (18). Clinically, Carstens and Correa de Sampaio et al. were the first to demonstrate the prognostic potential of spatial quantifications of T cells in proximity to cancer cells (15). Mass Spectrometry imaging has also been used to localize molecules of various sizes and proteins. The field continues to expand, now applying metabolic and functional markers in addition to sub-cellular localization to access inter-tumoral heterogeneity, co-localization, and therapeutic response (27–31).

Spatial transcriptomics profiles gene expression within tissue sections, connecting molecular details with histological context. Untargeted spatial transcriptomics is an approach designed to capture and analyze the expression of all mRNA transcripts within a tissue while preserving their spatial context. Unlike targeted methods that focus on predefined gene panels, untargeted techniques leverage next-generation sequencing (NGS), similar to scRNA-seq, to comprehensively profile the entire transcriptome. A key feature of this approach is the incorporation of spatial barcodes, which enable the assignment of each transcript to its original tissue coordinates, allowing for mapping of gene expression patterns across spatially distinct regions (32–34). These methods aide in developing spatial tissue atlases and the characterization of distinct tumor microenvironments, including tumor interfaces and tertiary lymphoid structures (35–38). These techniques can be variations of laser capture microdissection and use UV-photo-cleavable spatial targeting or barcoding (24, 39–41). Furthermore, these spatial transcriptomics and scRNA-seq are complementary approaches that, when integrated, provide a comprehensive view of cellular identity within the spatial architecture of tissues, providing insights into cancer progression and therapeutic targets (42–44). While scRNA-seq offers high-resolution transcriptomic data and the ability to identify novel cell subtypes, spatial transcriptomics retains the positional information of cells within their native microenvironment (45). Integration of these techniques is commonly achieved through two main strategies: deconvolution and mapping. Deconvolution aims to resolve distinct cellular subpopulations within each spatial transcriptomics capture spot by leveraging scRNA-seq data. Mapping involves two key components: assigning scRNA-seq defined cell subtypes to individual cells within high-plex RNA imaging maps and localizing each scRNA-seq cell to a specific anatomical region within the tissue. Such analyses offer spatial resolution to help predict ligand–receptor interactions inferred from scRNA-seq data, enabling insights into cell–cell communication within the tissue microenvironment (46–54). Continuing innovations in these areas have increased the imaging resolution to sub-cellular levels to allow single-cell resolution within the native tissue structure (55, 56).

Targeted spatial transcriptomics often uses fluorescence *in situ* hybridization (FISH) to identify single RNA molecules (57–60). Its benefits include sub-cellular resolution and the ability to enhance the capture of desired transcripts and isoforms that could be lost to dropout from unbiased techniques. Spatial Perturb-Seq combines untargeted whole transcriptomics with targeted CRISPR barcodes to assess the CRISPR screening and enables single-cell resolution analysis of genetic perturbations within intact tissue architecture. As a result, this method reveals both cell-autonomous and non-cell-autonomous effects while providing key insights into cellular crosstalk that are often missed by conventional dissociated single-cell sequencing (61). These methods effectively map tissue architecture and cellular clonality, serving as valuable tools to study TME heterogeneity (62).

Spatial omics platforms have recently integrated the evaluation of various molecule types, termed multi omics. These techniques enable protein confirmation of transcriptional signatures and assess tissue architecture’s influence on protein translation and cell signaling (63). Other multiomic techniques combine 3D structural rendering of serial sections with spatial targeted whole exome sequences, mass spectrometry imaging, spatial proteomics, and spatial transcriptomics (64, 65). The recent advancement of statistically principled and artificial intelligence based computational tools in spatial omics enabled the study of tissue organization by integrating spatial information with molecular profiling, allowing researchers to explore how the spatial arrangement of cells and molecular markers contributes to cancer biological functions in TME. One of the principle tools is focused on spatially variable gene analysis to identify genes that exhibit spatial heterogeneity in their expression in spatial domain, which is essential for understanding the functionality within tissues (66–68). In the next step, spatially varying network estimation focuses on regulation pattern at a molecular level and identification of transcription factors or key biomarkers which are potential targets for immunotherapy (69–71). Cell-type annotation and cluster analysis based spatial region identification help to decipher the complex cellular architecture of tissues by associating molecular profiles with specific cell types and spatial regions within TME (48, 72–74). The integration of scRNA-seq and spatial omics data will harness information from multiple omics layers to achieve higher precision in inferring clinical biomarkers in TME for immunotherapy based therapy intervention (75, 76). These innovations are being applied to understanding the TME and have identified structural and expressional heterogeneity far outside what has been previously understood. Overall, spatial assessments of the TME have contributed to discovering new biomarkers, predictors of clinical and therapeutic outcomes, novel phenotypes, and standards for tumor grading (21, 77–81). These modalities reveal the locations and triggers of phenotypes but not their onset or later progression. To fully grasp spatial TME biology and optimize its clinical applications, it is crucial to determine the specific timing and duration of these changes.

## Assessing time

Cross-sectional studies analyze gene expression in multiple samples at a singular time point across different conditions, treatments, disease stages, or patient cohorts. They are snapshots of molecular and cellular activity, making them valuable for identifying differentially expressed genes, discovering biomarkers, and understanding disease mechanisms (82). Longitudinal sampling expands cross-sectional analysis by tracking cellular states and gene expression changes in the same individuals or samples across different time points. It involves repeated sampling and profiling of experimental systems at multiple time points to capture temporal changes. Longitudinal labeling, on the other hand, labels cells or molecules at distinct time points but collects the data in a single snapshot. By integrating temporal data, longitudinal studies can identify the onset of genetic expression as well as trace the expression trajectory of dynamic biological systems, including tumor evolution, immune responses, and disease progression (83).

### Computational extrapolation of single cell multi-omics

While longitudinal sampling offers direct insight into temporal dynamics, computational approaches have emerged as effective alternatives or complements, enabling the inference of cellular trajectories and gene expression changes over time using static or limited time-point single-cell datasets. These methods have been adapted to appreciate cellular dynamics through computational extrapolations with or without longitudinal sampling.

Purely computational approaches of scRNA-seq datasets include monocle3 and wishbone used for “pseudotime trajectory analysis” which extrapolates the relationship of the transcriptional profiles of each cell as if one cell evolved from another using a single starting point (84, 85). These tools have been used in many publications to suggest clonal evolution and differentiation patterns of tumors (86–88). They have also been combined with longitudinal samples to confirm trajectory outputs (89).

Computational approaches are strengthened by incorporating longitudinal samples as time-point ground-truth within the analysis. TDEseq utilizes non-parametric statistical models to account for batch effects and confounding factors to enhance the detection of gene expression changes across multiple time-points (90). Additionally, Ramazzotti et al. introduced a Longitudinal Analysis of Cancer Evolution (LACE) for reconstructing longitudinal clonal trees that depict tumor evolution. This approach employed longitudinal single-cell somatic mutation profiles from tumor samples to monitor cancer evolution and intra-tumor heterogeneity over time and was used to evaluate therapeutic effectiveness and resistant sub-clone detection (91). The Time Series Analysis (TiSA) pipeline is another computational tool focusing on integrating data from multiple time-points to capture the temporal progression of cellular states, trajectories, and interactions. Its significance lies in its ability to address common challenges in biological data analysis, such as handling few replicates and uneven sampling within experimental groups. It employs a novel clustering method called PART, which identifies small genomic clusters for independent analysis, enhancing the biological interpretation of data through functional enrichment analysis (83, 92, 93). Its ability to investigate individual time points without requiring additional analyses highlights its robustness and flexibility, making it accessible to both clinicians and biological researchers. Taken together, the computational methods integrating longitudinal sampling are feasible tools for understanding the temporal progression of disease. While the aforementioned techniques help study temporal shifts in the TME, innovative strategies are required to definitively track TME cells and their dynamic evolution over time.

### Tracking cell dynamics

Longitudinal sampling has proven a powerful tool to extrapolate cellular dynamics; however, it does not study the same cells overtime. To overcome this limitation, the field has developed methods to label cells across time in a way that can be separated out at a common endpoint.

#### Lineage tracing

Lineage tracing is a technique for understanding cell differentiation and development by tracking the progeny of specific cells over time. This method involves labeling cells with fluorescent markers, such as conditional tracing with genetically controlled recombinases, or molecular barcodes, like CRISPR-Cas9, to follow the lineage and fate of these cells as they divide and differentiate (3, 94). Flow cytometry and multi-omics approaches can be used to detect and quantify these fluorescent markers and barcodes, respectively, providing a high-throughput means of analyzing cell populations (95). For example, in the study of minimal residual disease (MRD) in B-lineage acute lymphoblastic leukemia, flow cytometric analysis to track lineage tracing of leukemic cells predicted relapse and survival outcomes (96). A multicolor lineage tracing approach in colon cancer revealed clonal architecture and dynamics, showing a differentiation gradient from the tumor margin (marked by nuclear β-catenin and FRA1) to the center (marked by CK20 and GLUT1), where clonal competition indicated stable driver mutation profiles, suggesting spatial rather than genetic influences (89). Advances in lineage tracing have also incorporated CRISPR-based technologies, which augment the integration of unique genetic barcodes into cells to enable detailed reconstruction of cell lineages and developmental hierarchies. This approach, exemplified by the GESTALT method, enables the generation of thousands of unique barcodes, allowing us to appreciate tissue development and disease progression (97). Yang et al. combined lineage tracing with scRNA-seq to demonstrate that tumor evolution follows distinct phylogenetic trajectories, driven by genetic mutations, leading to enhanced cellular plasticity, correlating to more aggressive tumor states (98). Another study by Nadalin et al. used a similar method to highlight that tumor-initiating clones share a common chromatin priming state associated with specific transcriptional and epigenetic profiles using a Perturb-Seq guide barcode library (99).

Lineage tracing offers key strengths, like selectively and precisely labeling cells with genetic tools to track their progeny, helping us understand cell fate and tissue homeostasis, including how clonal expansion drives cancer progression. However, it can be limited by the need for specific markers, off-target effects and multiple double stranded breaks resulting in genotoxicity, limited targetable tissues, and single time point tracing (100).

#### Reporters

Reporter systems are molecular tools that reveal a cell’s functional state by linking a reporter gene to a regulatory sequence of interest, allowing quantification of active biological events. The reporter gene encodes a measurable product, such as a fluorescent protein (e.g., GFP, RFP), enzyme (e.g., luciferase), or chromogenic marker (e.g., X-gal staining), producing a detectable signal under specific conditions (101). Reporter systems can be classified as constitutive or inducible based on gene expression regulation. Constitutive reporters are often used to ensure successful transduction/transfection of cells while inducible reporters can be turned on and/or off and are usually used to study expression of a specific gene under specific conditions (102). Jun-Seo et al. developed a HeLa-Mx2 reporter cell line, containing a luciferase gene under the Mx2 promoter, an expression target of IFN-α, allowing precise quantification of IFN-α activity via luminescence (103). Massara et al. used a dual fluorescent reporter system with GFP to trace cancer cells and a modified, secreted mCherry protein (sLP-mCherry) endocytosed by CD206^+^ macrophages to track tumor-to-host communication in brain metastasis (104). Reporters can be combined with lineage tracers to capture both past and active expression of target molecules. For example, Perelli et al. developed a novel combined Cre- and Flipase-responsive reporter to trace the evolution of epithelial to mesenchymal transition in a model of pancreatic cancer. The cancer cells expressed tdTomato only if both the transforming Cre-expressing adenovirus and vimentin have been expressed. A FLEX-GFP Vimentin reporter also recorded cells actively expressing vimentin, so cells that have ever expressed vimentin were red while cells that actively expressed vimentin were both red and green (105). The authors also used Vimentin^FLEX^ mice to enable Cre-dependent, tumor-specific ablation of proliferating mesenchymal-state cancer cells upon ganciclovir treatment. This system permitted precise, time- and tissue-restricted elimination of mesenchymal cancer cells, revealing their essential role in sustaining both primary and metastatic tumor growth (105). Reporter systems enable real-time, non-invasive detection with high sensitivity and quantitative analysis while being easily adaptable to various animal models. However, challenges can come from the requirement of genetic engineering and the limitations of fluorescent tracers, such as endogenous cellular autofluorescence interfering with reporter signal and photobleaching/signal decay during prolonged light exposure.

#### Longitudinal labeling

Another means of tracking cell fate across time is adding a tag to cells at specific time points via longitudinal labeling. Common techniques use genetic approaches similar to lineage tracing and reporters but with an inducible genetic event as well as fluorescent or isotope additives that can be incorporated within specific time windows.

The classic pulse-chase experiments are versions of longitudinal radio-labeling of RNA and proteins that measure synthesis and degradation by tracking the incorporation (pulse) and subsequent decay (chase) of labeled nucleotides/amino acids. This technique has been widely used in cancer biology to study concepts ranging from degradation of tumor suppressors, protein folding and trafficking, and cellular metabolism (106–109). For example, CD36, a scavenger receptor, facilitates metabolic crosstalk between macrophages and cancer cells by uptaking tumor cell-derived extracellular vesicles enriched in long-chain fatty acids, which enhances their tumor-promoting potential and contributes to a pro-metastatic environment (109). In another study, a pulse-chase experiment with EU-labeled RNA revealed key effects of MYC in breast cancer cells. It showed that MYC boosts both RNA synthesis and decay rates. This increased RNA turnover induces oncogenic stress leading to cell death, highlighting its role as a potential therapeutic target (108).

Computationally, the open source pulseR package provides tools for analyzing RNA turnover from the experiments, supporting various experimental designs and addressing potential labeling biases (110). This method allows researchers to study physiology without overexpressing molecules, offering an efficient way to explore multi-step signaling pathways while accurately reflecting natural conditions due to minimal cell disturbances. However, pulse-chase struggles with molecules that have long half-lives (80, 90, 91). Another critical factor is the pulse duration, as longer pulses can disrupt RNAs and proteins (92).

Stable Isotope Labeling by Amino acid in Culture (SILAC) is another method to examine metabolic changes across the proteome. It monitors protein synthesis, degradation, and turnover using stable, non-radiolabeled isotopes detected by mass spectrometry (111). This technique is increasingly used in cancer research to investigate tumor development, progression, and therapy resistance. For example, Kim et al. found that oxaliplatin-resistant pancreatic cancer cell lines had higher levels of MARCKS and pAKT proteins than parental cells. Since MARCKS activates the PI3K/AKT pathway, it might contribute to resistance (112). Standard SILAC is limited to dividing cells in culture, but new advances have shown *in vivo* SILAC mouse models (113, 114).

A parallel *in vivo* strategy is the KikGR (Kikume green -red) mouse model used to map cell fate, track cell lineage, and live image cell dynamics. KikGR is a photoconvertible green-to-red fluorescent protein which converts from green to red upon exposure to violet light, allowing researchers to track the movement of labeled cells. It is expressed in a variety of cell types, including dendritic cells, osteoclast precursors, and tumor-infiltrating monocytes (115). Moriya et al. used KikGR photoconvertible reporter mice to demonstrate that immunogenic tumor cell death promotes the migration of tumor-infiltrating dendritic cells (Ti-DCs) to draining lymph nodes where they initiate effective CD8^+^ T cell responses. By photoconverting Ti-DCs *in situ*, they showed that phagocytosis of dying tumor cells triggered DC emigration through HMGB1-TLR4 and ATP-P2X7 signaling, ultimately enhancing memory precursor CD8^+^ T cell formation and suppressing secondary tumor growth in a dendritic cell-dependent manner (116).

Zman-seq is another iteration of longitudinal labeling that uses fluorescent antibodies. Named after the Hebrew word for time, Zman-seq applies both fluorescence-activated cell sorting (FACS) and scRNA-seq to resolve cell-state transitions and molecular signaling networks over time. To do this, antibodies for CD45 are injected intravenously into mice every 12 hours beginning 60 hours before endpoint. Each time point and antibody is associated with its own fluorophore to mark when specific cells were in circulation. Upon tumor collection, CD45^+^ cells are sorted by tumor exposure time according to the fluorophores they expressed. The sorted immune cells are then analyzed via scRNA-seq to extrapolate how long they were in the tumor and how that continuous tumor exposure time influences gene expression, transcription factor levels, ligand interactions, and signal senders and receivers. All these data can be used to resolve the moment immune cells become dysfunctional or immunosuppressive in the tumor. For example, its initial use revealed the temporal dynamics of immune cells in the glioblastoma TME. They found that monocyte to TAM transition correlates with tumor exposure time and NK cells became dysfunctional the longer they were in the tumor. Trem2 was also identified as a good immunotherapeutic target for redirecting TAM differentiation towards an anti-tumor phenotype. As a new method, Zman-seq has not yet been applied to many studies, but its implementation would capture single immune cell dynamics in real-time to inform how TME shape immune cell function. If paired with spatial transcriptomics and imaging, it could show which adjacent cells specifically contributed to these changes. Limitations such as limited fluorophores, fading signals, and recyclable epitopes can constrain its applications; however, Zman-seq proves a feasible modality for identifying the specific identity trajectories immune cells experience upon entering the TME (117).

Overall, these temporal techniques serve as potent tools for studying infiltration, cell fate, and function. They can be limited by label efficiency and stability, computational limitations, downstream data analysis, and heterogeneity in large populations. Still, temporal analysis remains vital for elucidating the changes in the TME. Moreover, by incorporating these temporal analyses with a spatial dimension, researchers can achieve a thorough perspective of how time and space collectively influence cell fate and function.

## Assessing time and space

The previously mentioned methods are useful for assessing temporal or the spatial context of a change, but they do not provide both. To assess tissue dynamics, spatial methods should be paired with real-time assays. This would create a more complete picture of the complexity of biology that single time point and non-spatial methods cannot attain. The following section discusses methods of real-time investigation that incorporate space and their future applications in cancer biology.

### Single-molecule/cell real-time imaging

Real-time imaging can assess the secretion of single molecules. One method, the interferometric detection of scattered light (iSCAT), tracks single particle release from single cells. In iSCAT, a cell secretes a protein within a detection field of view that appears as diffraction-limited shadows. The darkness of the shadow indicates the amplitude of the electromagnetic field that was scattered by the protein, thereby quantifying the presence and spread of protein secreted with single-cell resolution (118). iSCAT has already assessed secretion of IgG from Laz388 cells derived from B lymphocytes immortalized by Epstein-Barr Virus (118) and could be similarly used to assess the secretion of IgG and cytotoxic granules from TME-exposed B, NK, and T cells to assess lymphocyte exhaustion.

Another label-free high-throughput method uses hyperspectral photonic crystal resonant technology (PCRT) to map a gradient of protein secreted from single cells and assesses binding capacity of molecules. In PCRT, capture molecules, like antibodies, bind the target protein to cause a refractive index change and create the mapped gradient of protein secretions. These maps can be from single or multiple cells if run in parallel in a large field of view, providing the ability to image protein secretion from multiple single cells at once in real-time. This method has been applied to understanding the effects of cancer cell secretions on other populations by mapping platelet-mediated regulation and secretion of thrombopoietin from a human hepatocyte carcinoma cell line (119) and could be applied in TME research. However, PCRT has low throughput, does not specifically quantify the amount of secreted protein, can be influenced by non-specific binding, and has low spatial resolution (119).

Instead, similar secretion range analyses done via a label-free nanoplasmonic microwell array imaging system can create 4D (x, y, intensity, time) quantifiable spatiotemporal secretion maps from single-cells. This modality uses machine-learning cell tracking to capture cellular morphology and motility as it maps secretion. It has already been used to characterize antibody secretion from human hybridoma cells (120). Despite a small imaging window, future directions of this technique aim to add multiplexing capabilities to detect multiple secreted biomolecules at a time in addition to monitoring more than one cell type at a time. Such innovations would contribute to studies on cancer and immune cell interactions, such as analyzing how one immune-cell based cytokine could influence the morphology of cancer cell lines from epithelial to mesenchymal and tracing the changed cytokines the cancer cell releases upon morphological changes. If adapted to track more than one cell type at a time, label-free nanoplasmonic microwell array imaging system could record immune cells in conjunction with the surrounding cancer cells, improving the ability of capturing cell-cell signaling interactions and their consequential influence on the TME in real-time (120).

Another imaging method, total internal reflection fluorescence microscopy (TIRFM), does require a label and specialized equipment but can provide real-time and high throughput imaging of specific protein secretion. In this case, the fluorophore on the target is only excited if it is a specific distance from the cell (121). TIRFM has been used to assess immune cell secretion such as IL-1β from non-classical monocytes and revealed the order in which monocytes lost their cell membranes and secreted IL-1β (196). F-actin patches have been analyzed with TIRFM (121), and it has also been applied to studying the levels of apoptotic and necrotic death that Campthothecin and Cisplatin induces in breast cancer cells (122). As TIRFM can observe cell secretion and other activities near cell membrane surfaces, it would be a strong candidate for capturing cytotoxic immune cell activity in TME such as CD8+ T cell or NK cell release of Granzyme B to kill cancer cells or even record immune escape interactions such as conjunction of PD-1 and PD-L1.

These *in situ* tools provide means to assess signaling and secretion patterns of cells in the TME. Each method has its own limitations depending on label-need, downstream analysis, and required equipment, but all are robust ways of capturing cell secretion in real-time. For a detailed analysis of these methods with other TME models, see this review (123). Combining these single-cell based signaling assays with more robust TME models would provide improved translational capabilities to current standard lab practices.

### Multi-cell real-time imaging and biosensors

#### Fluorescent biosensors

Fluorescent biosensors track cellular processes as they occur by measuring the presence of a specific molecule in a mixture in real-time. When this ligand binds to the sensor, the signal induces a quantifiable physical change corresponding to the amount of the target in a sample (urine, saliva, blood, plasma, etc.) (124). Fluorescence-based, genetically encodable biosensors provide information for events such as protein phosphorylation, metabolism, neurotransmission, hormone analysis, DNA and toxin detection, medical diagnoses, and drug delivery and discovery; they are also used in multiple research fields, including tumor biology (124–131). Fluorescence resonance energy transfer (FRET) is a biosensor with a fluorophore/quencher pair that conjugate to any target in the vicinity and trigger a fluorescent signal that can be imaged (123). Using FRET, cancer studies have highlighted specific peptide interactions, spatiotemporally visualized signaling activity, and tested therapies involving apoptosis and immune checkpoint inhibitors (132–137). FRET has detected the amount of lactate in a single cancer cell (130) and visualized kinase signaling activity and how specific drugs induce apoptosis in cancer cells (132–135). Time resolved FRET tested small molecule inhibition of LAG-3 on T cells in relation to FGL1 on human cancers (137) while nanosensors identified specific exosomes to try to diagnose cancer earlier (136). As with most fluorescent modalities, signal overlap and tissue penetrance can pose problems with analysis, but there are workarounds. Even so, as customizable, quantifiable, non-invasive, and high-resolution ways to analyze live-cell signaling and biomolecule presence in real-time, biosensors prove to be a useful tool in studying dynamics within the TME. When combined with spatial and transcriptomic data, these analyses could characterize the heterogeneity of TMEs across time.

#### Bioluminescence imaging

Bioluminescence imaging (BLI) is another non-invasive direct imaging technique designed to measure luminescence in genetically modified cells to trace their activity (138). BLI works by genetically engineering cells to express a luciferase enzyme reporter which, when bound to a specific added substrate, emits recordable light (139). Originally, the firefly luciferase gene, *luc*, was added into a virus’ genome and used to detect infected cells in culture and in mouse tissue, creating a method for tracking the spread of viral infection (140). Since then, luciferase reporters have expanded to include other insect species, marine organisms, whole body cancer progression models, immune infiltration, and immunotherapy efficacy assays; they even have multiplexing ability (139, 141–143). For example, a dual color luciferase reporter mouse identified “hot” *vs* “cold” pancreatic cancer tumors based on T cell infiltration and activation states (142). Dual luciferase reporters tracked tumor cells and T cells with and without chimeric antigen receptors (CAR) to see how CAR addition increases tumor killing capacity of T cells *in vitro* and *in vivo* (143). Other applications of BLI in the TME could include longitudinal tumor growth, attempts at capturing circulating cancer cells in the blood stream, or immune cell secretion of cytotoxic granules in a tumor as seen done by Chen et al. to help track checkpoint inhibitor efficacy (144). BLI’s effectiveness can be limited by tissue penetration, spectral overlap, spatial resolution, luciferase kinetics, and its dependency on substrate (145). While BLI is applied in longitudinal studies, these snapshots of luciferase activity are captured in real-time, allowing the collection of biologically relevant data that can be followed in the same specimen. BLI is commonly used in TME analysis already, and it is often used in conjunction with real-time imaging practices as we will discuss. By providing a relatively simple yet specific way of seeing more than one cell population interact at a time, BLI applications in the TME allow researchers to watch cell dynamics in environments of varying size.

#### Tumor on a chip

Recent advances in organoid and microfluidics have created tumor-on-a-chip (TOC) to model the three-dimensional complexity of TME *in vitro*. TOC has captured components of TME including cancer cells, vasculature, stromal cells, immune cells, and ECM in addition to heterogeneous metabolic factors like oxygen, pH, nutrient gradients, and growth factors (123). These features can self-organize or be designed to mimic the specific TME of humans or mice, improving upon standard *in vitro* cell cultures and 3D spheroids/organoids (123, 146, 147). For instance, vascularizing TOC with microfluidics successfully models drug delivery, biochemical diffusion, and metastatic dynamics (148–151). The benefits of being an *in vitro* system allow for fine-tuned control of the environmental conditions and gene expression of the constituent members as well as the use of standard cell proliferation, viability, apoptosis, migration, and secretory assays. TOC captures spatiotemporal heterogeneity of tumors more than a cell line and has plenty of potential integration with omics. Therefore, TOC has high translatability to *in vivo* studies while providing clear applications to personalized medicine. It has been used to mimic bone marrow structure to compare healthy and leukemic TME (146) and model breast cancer cell metastasis (151). One study used TOC to model the TME of glioblastoma including immunosuppressive tumor associated macrophage modulation and cytotoxic T cell response to anti-PD-1 therapy (147). While TOC cannot capture the systemic influences seen *in vivo* and has a simplified heterogeneity, it remains a model in which applying the above real-time and single-cell assays to TOC could provide higher resolution in current TME *in vitro* studies. Other uses for TOC could be tracking immune cell homing ligands secreted by the tumor to get a three-dimensional map of chemokine concentrations over time or even immune cell movement through the TOC itself. It could potentially be used to highlight heterogeneity and minimal amount of MHC-I presentation by cancer cells to immune cells more robustly than standard cell lines. TOC creates an opportunity to study tumor heterogeneity *in vitro*; however, nothing is more translatable than being able to record cellular activity in the animal as it is happening. Intravital imaging allows for exactly that.

### Intravital imaging

Intravital imaging (IVI), also called intravital microscopy, incorporates many of the modalities we have discussed including BLI and lineage tracing to record cell movement and interactions inside the live animal as they occur. IVI can be used to measure acute or chronic changes with repeated imaging of the same animal, gaining a comprehensive depiction of live cell processes (152). For acute studies, IVI focuses on thinner tissues that light can pass through for direct imaging such as the ear, cremaster muscle, or salivary gland of a mouse. However, for chronic studies, the entire organ or its surface can be externalized for imaging. Alternatively, an imaging window or chamber is installed directly into the mouse for up to a month of imaging (152). These windows have been used to study a wide array of organs including the brain, skin vasculature, lymph nodes, liver, spleen, pancreas, small intestine, kidneys, lungs, ovaries, and long bone (152–162). The cranial imaging window can be used for long-term brain imaging though open-skull or thinned skull windows. One study used this model to record glioblastoma tumor cell migration into the tumor core, border, or invasive front (155). Abdominal imaging windows capture any organ in the abdominal cavity and can image for up to a month. Using these windows, scientists have tracked mouse colorectal cancer metastasis to the liver and recorded pre-micro and micro-metastases (154). Another study quantified pancreatic tumor cell hypoxia and its spatial relationship with collagen and microvasculature architecture (163). Lung imaging windows have visualized single tumor cells interacting with macrophages and monocytes in lung vasculature (157). Other applications recorded all steps of tumor metastasis and seeding to the lung and even tracked neutrophils in pulmonary capillaries working as defenders against pathogens (158, 159). Dorsal skinfold chambers allow for imaging access of muscle cells and mouse skin and have assessed solid tumor interactions with bone in a miniaturized tissue-engineered bone construct (160). Other methods of IVI include through the body cavity or by externalizing the organ. This strategy has visualized resident macrophage cloaking in response to cell injury to prevent neutrophil-mediated inflammatory damage (161). It has also been used to assess granulocyte monocyte progenitor populations in the spleen of cancer bearing mice, finding that the spleen serves as a source of tumor associated macrophages and neutrophils (162). When these organ visualization methods are used with cell-tracking such as BLI, lineage tracing, or reporters, IVI easily captures real-time cellular activity of cancer, stromal, and immune components of the TME in a live animal (163, 164).

Limitations of IVI include imaging depth penetration dependent on the type of microscopy used, light scattering properties of the imaged tissue, and limited number of fluorophores the camera can simultaneously capture (152). Immobilization could induce shear force on tissue, and many IVI models have to use immunodeficient mice to avoid reactions to the cameras. Imaging time frame can also be a limiting factor, but downstream fixing, sectioning, and staining of the tissue in the imaging area can be correlated with the videos taken with IVI for confirmation and deeper analysis of the end-point recording (156). Image analysis itself can also be a bottleneck due to the densely associated cells in tissues and large data files. Machine learning algorithms have become more common to assist with IVI analysis, especially time-lapse recordings (156). Despite these limitations, IVI remains a comprehensive method of understanding the dynamics of cancer cells and the TME in their natural environment, expanding our knowledge of tumor development and treatment response. More details about intravital imaging methods and applications can be found in these reviews (152, 153, 156, 164, 165).

IVI lets researchers look into a live animal and record biological processes as they happen, so events like cell movement and cytokine release can be visualized in real-time. This advancement in biological research will expand translatability to cancer diagnostics and treatment. With biomedical imaging becoming a major tool for cancer identification and therapy, increasing IVI studies for potential incorporation with diagnostic and therapeutic imaging would provide an efficient axis for elevating research from mice to humans.

### Diagnostic and translational imaging

Biomedical imaging is of prime importance in comprehensive cancer care. It offers numerous benefits like real time surveillance, non-invasive access to the tissue, and the ability to operate over a wide spectrum of spatial and temporal levels involved in the biological system (166). Ultrasound, Computed Tomography (CT), and Magnetic Resonance Imaging (MRI), and radiotheranostics are major techniques for biomedical imaging, each offering unique advantages and information (**Table 1**). These methodologies are also available in the pre-clinical setting, thereby allowing mechanistic studies in model systems that can be translated into the clinic.

**Table 1:** Strengths, limitations, and TME applications of diagnostic and translational imaging.

Method	Strengths	Limitations	Applications in TME
*Ultrasound*	• Utilizes sound waves to create images of internal body structures • Non-invasive • Real-time imaging • Safe, does not use ionizing radiations • Cost effective	• Sound waves do not pass through bones and air • Might not capture deep tissues in the body	• Routine cancer screenings and lesion characterization in organs like the liver, urogenital tract, and soft tissues (167) • Early breast cancer detection (168, 169) • Used microbubbles linked to molecularly-targeted ligands that bind to specific tumor markers, enhancing contrast and facilitating the identification of cancerous tissues (170)
*Magnetic Resonance Imaging (MRI)*	• Non-invasive imaging to produce 3-D anatomical images using magnets and radio waves • Contrast agents enhance ability to detect specific cellular/subcellular events • Non-invasive • No ionizing radiations	• Uses very strong magnetic field, thereby excluding people with any form of implants, especially those containing iron • Expensive and more time consuming than CT	• Agents can be targeted to specific receptors or molecules, allowing for detailed imaging of tissues like myocardium, atherosclerotic plaques, or tumors (171–173) • Dynamically monitors changes in tumor vascular structures and immune responses (174) • Integrates with photoacoustic imaging (MR/PA), enhancing its utility in assessing therapeutic responses (174)
*Computed Tomography (CT)*	• Utilizes x-rays to produce high-resolution cross-sectional images of the body • CT can create 2D or 3D images, which can be rotated to detect the issue • Quick scans (less than 1 minute)	• Exposes patients to more radiation than x-rays • Less proficient than MRI to detect soft tissues • Contrast agents can cause allergic reactions and lethargy in patients • Transplants and metal objects can produce artifacts • Not suitable for pregnant women	• Evaluates, stages, and monitors cancer with intravenous and oral contrast agents enhancing assessment accuracy (175, 176) • Screening for lung cancer using low-dose CT in individuals with smoking history (177) • Colonoscopy combined with CT (virtual colonoscopy) to detect large colorectal polyps and tumors (178)
*Positron Emission Tomography (PET)*	• Generates 3D functional images of the body by detecting gamma rays emitted from positron-emitting radiotracers (fluorodeoxyglucose, FDG). • Non-invasive • Often combined with CT or MRI to enhance spatial resolution and provide anatomical context	• Low signal to noise ratio can lead to noisy images and/or inaccurate detection of very small lesions and subtle changes in tracer uptake • Spatial resolution of 4.5mm can lead to artifacts and issues in detecting small lesions	• Diagnoses tumors, assesses metastases, and evaluates treatment responses (179) • Measures molecular properties of diseases crucial for prognosis, therapy selection, and monitoring early and long-term responses to treatment (180)
*Radiotheranostics*	• Detects the presence of a target in patients before giving them treatment, separating responders from non-responders. • Consists of a radionuclide for diagnosis or therapy, a ligand or probe for a cancer-specific molecular target, and a chelator to link them • Real-time • Diagnostic and therapeutic functions • Precision targeting to minimize side effects	• Limited availability of suited radioisotopes • Discrepancy between optimal imaging and therapeutic ability of the agent • Requires precise calculation of the dose to be delivered to the target tissue using complex theranostic agents	• Targeted prostate specific membrane antigen in prostate cancer and NaI symporter in differentiated thyroid cancer (181) • Fibroblast activation protein has been targeted in multiple cancers (182) • CD20 has been a target for B cell non-Hodgkin lymphoma as well as CD45 in acute myeloid leukemia (182)

Biomedical imaging performed in the context of longitudinal imaging can inform the evolution and progression of disease. A study by Javadi et al. found that 80% of patients with pancreatic cancer experienced locoregional or distant recurrence within two years post-resection. Using imaging modalities like multi-detector CT (MDCT), PET/CT, and MRI, they determined that baseline MDCT is effective in detecting early lesions after resection (183). Another study indicates that FDG PET-CT provides better diagnostic accuracy than CT alone for detecting recurrent PDAC. However, evidence supporting routine radiologic surveillance post-resection is limited, requiring further research to optimize follow-up strategies (184). These longitudinal imaging studies are further enhanced by the collection of biological samples at the time of the longitudinal imaging, acting to confirm the tissue, cellular, and molecular components of the lower resolution imaging data.

### Computational tools for spatiotemporal omics

High-throughput sequencing techniques combine high-resolution spatial and temporal profiling to study the dynamic organization and biological processes within TME. Spatial alignment tools like MOSCOT and SLAT align the heterogeneous tissues across multiple time points (185, 186). MOSCOT was used to overcome the limitations in spatial transcriptomic data by integrating gene expression, protein abundance, and single-cell annotations to accurately characterize liver zonation and align large-scale tissue sections. This enabled the identification of central and portal veins, mapping of Kupffer cells, and construction of a consensus tissue view. On the other hand, SLAT accounts for non-rigid structural changes without manual annotations and offers scalability, adaptability, and real-time performance, making it a robust tool for diverse biological applications such as developmental mapping, in silico data enhancement, and cross-species comparisons. Spateo and stLearn integrate spatial and temporal expression for lineage tracing and cell fate inference during a biological process (187, 188). The joint integration of spatial and temporal dimension of omics layers is challenging from several perspectives such as alignment, tractability and resolution (189). Explainable AI (XAI) is also achieving broader recognition because of its ability to make complex machine learning models, especially deep learning, more transparent and interpretable—an essential step in understanding hidden patterns in spatiotemporal omics data (190). Among XAI tools, SHAP (Shapley Additive exPlanations) provides detailed information into how individual features influence a model’s prediction, offering both global and local interpretability (191). Grad-CAM (Gradient-weighted Class Activation Mapping), on the other hand, is a visual approach that highlights the regions of input images that drive convolutional neural network predictions, making it useful in spatial analyses (192). Though XAI is in its infancy in biomedical discipline, such methods help researchers build trust in AI models and better understand the underlying biology. Even though the tool development for spatiotemporal omics is in its early phase, it holds immense potential to revolutionize our understanding of complex biological processes by providing insights into the spatial and temporal dynamics of molecular interactions within tissues, paving the path for a new era of precision medicine (193, 194).

## Discussion

Tumor microenvironments and the specific dynamics of their components are highly complex, necessitating equally complex methods for their study. The components of the microenvironment are regulated by both space and time, and understanding both of those dimensions would allow for finely tuned windows of intervention in personalized care. Standard tissue sampling across longitudinal studies can extrapolate general TME evolution over time, but each sample is an isolated incident unable to track the lives of each cell across tumor progression. These methods can be enhanced with lineage tracing, barcoding, and active reporters to identify specific cell types undergoing fate changes. Spatiotemporal imaging of live cells *in vitro* and *in vivo* are ideal methods for recording cell activity in real-time and have the potential to be integrated with standard pre-clinical and clinical image technologies to translate these assessments to clinical practice. Overall, the addition of space and time to high-dimensional cellular analyses is becoming increasingly feasible for cancer biology and clinical translation.

### Future directions

The integration of spatiotemporal strategies, AI technology, and infrastructure readiness will shape the future of precision medicine to better capture the dynamic and contextual nature of cancer biology. Emerging tools that enable high-resolution, multiplexed, and real-time profiling of gene, protein, and metabolite expression across space and time will provide a deeper and better understanding into tissue architecture, cellular interactions, and disease progression. These advances will require robust, user-friendly computational platforms capable of handling high-dimensional, multimodal data. AI and machine learning modalities are powerful tools for interpreting these complex spatiotemporal dataset. Such analysis could help personalize clinical decision making once these technologies are supported by rigorous validation and standardized protocols. As spatiotemporal datasets continue to grow, comparison between diverse cohorts will enhance clinical relevance and reproducibility. Additionally, achieving spatiotemporal readiness—coordinating where and when therapies are manufactured, processed, and delivered—will be vital to ensure timely, cost-effective, and equitable access. Together, these strategies demand interdisciplinary collaboration and thoughtful infrastructure planning to realize the full promise of spatiotemporal precision medicine.

## Conclusion

To move beyond an *in medias res* understanding of tumor microenvironments, we must incorporate both spatial and temporal perspectives into our research frameworks. While spatial biology has delivered critical details into cellular localization and interactions, and temporal strategies have revealed dynamic changes in molecular activity, their integration offers a more complete and actionable picture of disease progression. By capturing how cell states, signaling pathways, and microenvironmental contexts evolve over time and space, spatiotemporal analysis enhances our ability to identify therapeutic windows, track disease trajectories, and inform precision interventions. As tools and methodologies continue to advance, embracing this four-dimensional approach will be essential to fully unfold the complexities of biology and improve patient outcomes.
